# Exploiting the Invasive Alga *Rugulopteryx okamurae* for the Synthesis of Metal Nanoparticles and an Investigation of Their Antioxidant Properties

**DOI:** 10.3390/md23120479

**Published:** 2025-12-15

**Authors:** Estefania Pereira Pinto, Noelia González-Ballesteros, María Carmen Rodríguez-Argüelles

**Affiliations:** Departamento de Química Inorgánica, Universidade de Vigo, 36310 Vigo, Spain; estefania.pereira.pinto@uvigo.es (E.P.P.);

**Keywords:** *Rugulopteryx okamurae*, AuNPs, AgNPs, PtNPs, green synthesis, antioxidant activity

## Abstract

The rapid spread of the invasive brown macroalga *Rugulopteryx okamurae* has caused severe ecological and economic damage along the European coasts. Efforts to mitigate its impact have been largely ineffective, highlighting the need for alternative strategies to valorise this invasive species. This study explores the use of *R. okamurae* aqueous extract (RO extract) as a natural reducing and stabilizing agent for the green synthesis of gold (Au@RO), silver (Ag@RO), and platinum (Pt@RO) nanoparticles. The synthesized nanoparticles were extensively characterized using ultraviolet–visible spectroscopy (UV-Vis), transmission electron microscopy (TEM), X-ray diffraction (XRD), zeta potential analysis, and Fourier-transform infrared spectroscopy (FTIR). The results confirmed the successful formation of spherical and stable nanoparticles. Furthermore, the antioxidant activity of the RO extract was determined before and after the synthesis of the nanoparticles by the determination of the reducing power, total phenolic content and 1,1-diphenyl-2-picryl-hydrazyl (DPPH) radical scavenging activity. Notably, Pt@RO showed the highest enhancement in antioxidant activity among the nanoparticles synthesized. The findings demonstrate that *R. okamurae* can be repurposed as a valuable bioresource for the environmentally friendly production of metal nanoparticles with promising applications.

## 1. Introduction

The brown macroalga *Rugulopteryx okamurae* (E.Y. Dawson) I.K. Hwang, W.J. Lee, and H.S. Kim, 2009, (Dictyotales, Phaeophyceae), native to the northwestern Pacific Ocean, has become a critical issue in the Mediterranean and Atlantic coasts, causing severe environmental and economic problems and being considered one of the most aggressive invaders reported in recent years [1]. Currently, this invasive species is spreading rapidly along the coasts of Europe and its potential for further expansion is expected to continue [2,3]. Efforts to control or eliminate *R. okamurae* have proven largely ineffective, underscoring the need to repurpose this invasive species for practical and beneficial uses [4].

Brown macroalgae are widely recognized as rich sources of structurally diverse bioactive compounds such as fucoidans, phlorotannins, and alginates [5], many of which exhibit remarkable biological activities including antiviral, anti-inflammatory, antimicrobial, and immunomodulatory properties [6]. These bioactive molecules not only confer valuable biological properties but also make brown algae ideal candidates for green nanotechnology applications, where their polysaccharides, phenolic compounds and pigments function as natural reducing and stabilizing agents in the synthesis of metal nanoparticles (MeNPs) [7].

Green synthesis methods for the production of nanomaterials offer several advantages over conventional chemical and physical approaches. The use of natural resources as stabilizing agents enables the elimination of toxic and/or expensive reagents and solvents, reduces waste generation, and allows for milder reaction conditions. These features make green synthesis a more sustainable and cost-effective alternative [8,9]. In contrast, traditional methods often require harsh reaction conditions, involve hazardous chemicals, and demand high energy input, factors that contribute to environmental pollution, health risks, and increased production costs [10].

In line with our ongoing research on the green synthesis of MeNPs using natural extracts [11], this study proposes the use of RO extract for the synthesis of gold (AuNPs), silver (AgNPs) and platinum (PtNPs) nanoparticles.

The unique physicochemical properties of AuNPs, AgNPs and PtNPs make them highly versatile and effective in a wide range of applications across various fields, including agriculture, environmental remediation, cosmetics, food processing, nanomedicine or catalysis [12,13,14]. Moreover, in several of these applications, the activity of the nanoparticles can be significantly enhanced through synergistic interactions with specific compounds present in algae. These interactions may improve stability, biocompatibility, or targeted activity, further expanding the potential of algae-mediated nanoparticle synthesis in sustainable nanotechnology [15].

For instance, green synthesized AgNPs have demonstrated antimicrobial activity against a wide range of bacteria and fungi, making them suitable for different applications, including wound healing or food packaging [16]. Among other applications, AuNPs have been investigated for their antitumoral, anti-inflammatory and antioxidant properties [17] while green synthesized platinum nanoparticles are highly valued for their exceptional catalytic efficiency and potential in cancer therapy [18,19].

This study bridges environmental valorization and biotechnology by exploring for the first time the potential of *R. okamurae* to synthesize AuNPs, AgNPs and PtNPs, while also characterizing their properties, including their antioxidant activity. By addressing a pressing ecological problem with a novel biotechnological solution, this work demonstrates how this invasive species can be transformed from an ecological threat into valuable resources.

## 2. Results and Discussion

### 2.1. Synthesis of Au@RO, Ag@RO and Pt@RO and Characterization by UV-Vis

**Figure 1:**
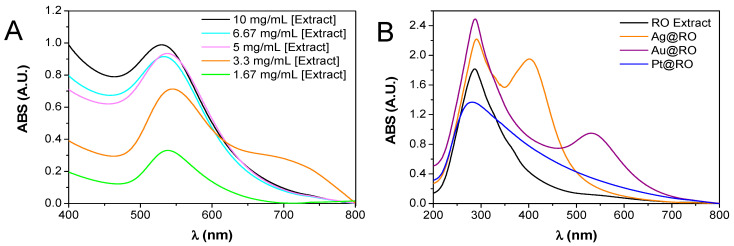
Ultraviolet–visible spectra analysis: (**A**) Different concentrations of RO extract with a fixed HAuCl_4_ concentration (0.42 mM). (**B**) Comparison of RO extract, Au@RO, Ag@RO and Pt@RO.

For the synthesis of Au@RO, the first assays were conducted with a fixed concentration of 0.42 mM of HAuCl_4_ and varying RO extract concentrations between 10 and 1.67 mg/mL at 30 °C for 24 h. After observing a color change in the reactions from pale yellow to burgundy, they were analyzed through ultraviolet–visible (UV-Vis) spectroscopy. As shown in Figure 1A, the SPR band in the UV-Vis spectra shifted slightly depending on the concentration of the RO extract. Higher extract concentrations (10, 6.67, and 5 mg/mL) produced intense bands at 530–538 nm, whereas lower concentrations (3.3 and 1.67 mg/mL) resulted in weaker and red-shifted bands around 545 nm. This blue shift observed at higher RO extract concentrations suggests the formation of smaller nanoparticles, consistent with literature reports on the size-dependent optical properties of metal nanoparticles [20]. Based on these results and previous studies [11,21], the experiments were extended by testing four different gold concentrations (0.17–0.5 mM) while maintaining RO extract concentrations of 10, 6.67, and 5 mg/mL (Supplementary Material, Figure S1). The optimal parameters for the following characterization of Au@RO were established using a RO extract concentration of 6.67 mg/mL and 0.33 mM of HAuCl_4_ (maximum absorbance, λ_max,_ 531 nm), with the reaction carried out for 24 h at 30 °C. By successfully completing the synthesis of Au@RO at 30 °C, the need for external heating was significantly reduced, which not only lowered energy consumption but also demonstrated the process’s efficiency under low-energy conditions.

For the synthesis of Ag@RO, the initial experiments were performed at 30 °C for 24 h, using a constant RO extract concentration of 10 mg/mL and varying concentrations of AgNO_3_ aqueous solutions ranging from 0.17 to 0.5 mM. However, at 30 °C, the experimental conditions did not promote the formation of AgNPs. A distinct amber color shift, and the appearance of the characteristic SPR band of AgNPs in the UV-Vis spectra was observed only after the solutions were heated to boiling temperature (100 °C), using a constant RO extract concentration (10 mg/mL) and varying AgNO_3_ concentrations between 0.1 and 0.5 mM. To determine the optimal time for achieving a narrower and more intense SPR band in the UV spectra, aliquots were collected every 30 min for up to 210 min. The highest intensity was observed with 10 mg/mL of RO extract and 0.17 mM of AgNO_3_ aqueous solution heated at 100 °C for 120 min (Figure 1B, λ_max_ 401 nm). Additional tests were conducted using lower extract concentrations (6.6, 5, and 3.3 mg/mL) while maintaining the same AgNO_3_ concentration (0.17 mM) to confirm that these conditions were indeed optimal.

Regarding the synthesis of Pt@RO, a series of reactions were carried out at 30 °C using a fixed concentration of RO extract (10 mg/mL) and varying platinum concentrations from 0.4 to 0.1 mM, but no reaction occurred. Subsequently, the same concentrations were used, but the reaction mixture was heated to boiling temperature (100 °C), resulting in a color change to dark brown when the highest platinum concentration (0.4 mM) was used. To further investigate the effect of RO extract concentration, different concentrations (6.6, 3.3, 2, 1, 0.5, and 0.1 mg/mL) were tested, and aliquots were taken at different time points for TEM characterization. The plasmon resonance band of Pt@RO appears in a region of high absorption by the RO extract, making it indistinguishable (Figure 1B, λ_max_ 280 nm). As indicated in the experimental section, the optimal reaction conditions for Pt@RO were determined to be 1 mg/mL of RO extract and 0.4 mM of platinum, with heating to boiling for 3 h.

### 2.2. Transmission Electron Microscopy Characterization of Au@RO, Ag@RO and Pt@RO

**Figure 2:**
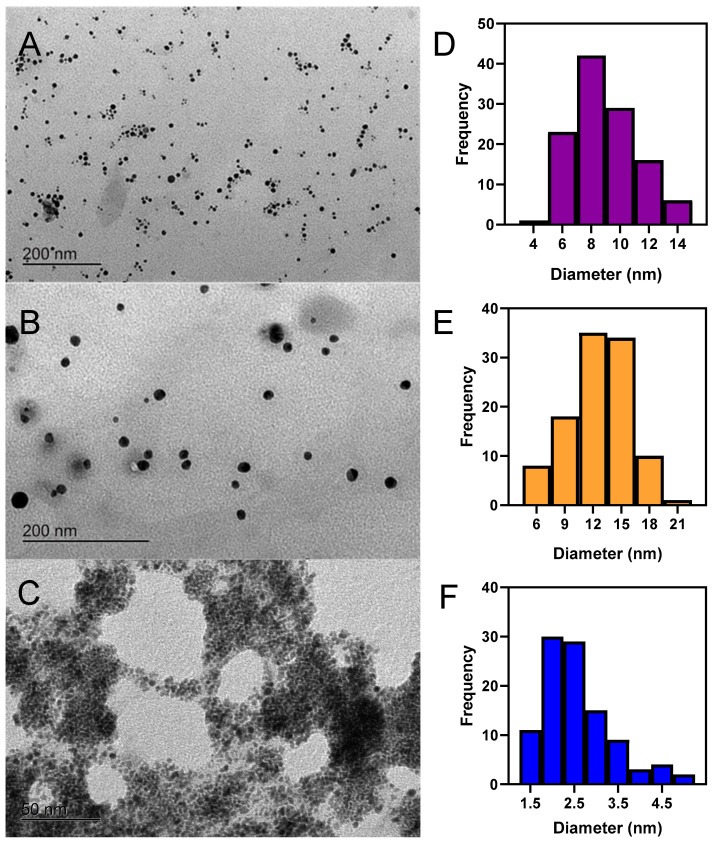
Transmission electron microscopy images and size distribution histograms of NPs: (**A**) TEM image and (**D**) size distribution histogram of Au@RO; (**B**) TEM image and (**E**) size distribution histogram of Ag@RO; (**C**) TEM image and (**F**) size distribution histogram of Pt@RO.

The size and morphology of the synthesized MeNPs under different conditions were characterized using Transmission Electron Microscopy (TEM). Some of these conditions resulted in a complex mixture of irregular shapes including hexagonal, quadrangular, and triangular shapes, in addition to the spherical ones (Figure S2). Figure 2A–C shows the TEM images obtained under the optimal synthesis conditions for Au@RO, Ag@RO and Pt@RO, respectively, illustrating their spherical shapes and uniform morphology achieved under the selected synthesis conditions. The diameters of over 100 NPs were measured to create a size distribution histogram, as shown in Figure 2D–F. The results indicate that Au@RO had an average diameter of 7.5 ± 2.3 nm, Ag@RO were slightly larger, with an average diameter of 12.9 ± 3.5 nm. This trend aligns with previous studies, which consistently report that AgNPs tend to be slightly larger than AuNPs synthesized from the same extract [11,22,23]. In the case of Pt@RO the smallest particles were obtained with a mean size of 2.6 ± 0.8 nm.

The size of the MeNPs obtained in this study aligns well with the results reported in previous works, although slight differences can be observed depending on the synthesis conditions and the seaweed extract used. As this represents the first report of MeNPs synthesized using RO extract, comparisons are most appropriately drawn with studies involving other brown macroalgae. Au@RO had an average diameter in a lower range than those reported in other studies using similar green synthesis methods. For example, AuNPs synthesized using *Sargassum muticum* were reported to have diameters ranging from 5 to 15 nm with a spherical shape [23,24]. Similarly, those synthesized with *Undaria pinnatifida* extracts also exhibited a spherical shape and a mean size of 6.8 ± 1.0 nm [11]. In contrast, AuNPs synthesized with *Spatoglossum asperum* exhibited cubic morphologies with an average size of 20 nm [25]. Even larger sizes, ranging from 53 to 67 nm, were observed after the synthesis of AuNPs from *Padina gymnospora*, which were also spherical in shape [26].

For Ag@RO, the average diameter of the NPs obtained in this study aligns with the size range reported for AgNPs synthesized from brown macroalgae in previous research [27]. Notably, Ag@RO exhibit dimensions comparable to those reported for AgNPs synthesized from various *Sargassum* species, which have shown notable variability in size and morphology. *Sargassum subrepandum* produced the smallest NPs, with sizes of 4.5 ± 1.2 nm, and exhibiting shapes that transitioned from spherical to cubic [28]. Slightly larger AgNPs were obtained from *Sargassum vulgare*, with sizes ranging from 6.90 to 16.97 nm and a predominantly spherical morphology [29]. *Sargassum polycystum* (formerly *Sargassum myriocystum*) AgNPs were well-dispersed with a hexagonal shape, having an average size of 20 ± 2.2 nm [30]. Other macroalgal species from the same Phaeophyceae class, such as *Polycladia myrica* (formerly *Cystoseira myrica*), yielded spherical AgNPs with sizes ranging from 8 to 15 nm [31]. For *U. pinnatifida*, the AgNPs exhibited a spherical shape with an average diameter of 14.1 ± 2.8 nm [11].

As for Pt@RO, the green synthesis of PtNPs using algae extracts remains largely unexplored. In the literature, only a few studies have reported the synthesis of PtNPs mediated by algae. Two of them used green micro and macroalgae species, *Botryococcus braunii* (86.96 nm) [32] and *Codium* sp. (15.75 ± 0.21 nm) [33]; two others employed the brown alga species *Padina gymnospora* obtaining two difference size range 10–60 nm [34] and 5–20 nm [35]; and one study used the red alga *Halymenia dilatata* (15 ± 1.7 nm) [36]. In all cases, the nanoparticles reported were larger than those obtained in this study.

### 2.3. Crystallinity Characterization of Au@RO, Ag@RO and Pt@RO by XRD and HRTEM

**Figure 3:**
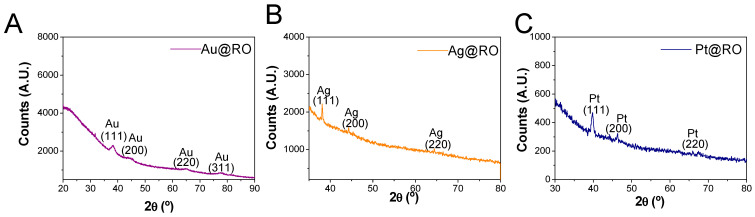
X-ray powder diffractogram of (**A**) Au@RO, (**B**) Ag@RO and (**C**) Pt@RO.

The crystalline structures of Au@RO, Ag@RO and Pt@RO were initially examined using X-ray diffraction (XRD) analysis, as shown in the diffractograms presented in Figure 3A–C, respectively. For the Au@RO sample, the obtained diffraction pattern was compared to the standard JCPDS powder diffraction reference card for gold (File No. 04-0784). The diffractogram exhibited four distinct peaks at 2θ positions of 38.15°, 44.83°, 65.10°, and 77.25°, corresponding to the Miller indices (111), (200), (220), and (311), characteristic of the face-centered cubic (FCC) structure of gold. Similarly, the diffractogram for Ag@RO displayed three peaks at 2θ positions of 38.10°, 44.27°, and 64.44°. Comparison to the JCPDS reference card for silver (File No. 04-0783) confirmed that these peaks correspond to the Miller indices (111), (200), and (220) of the FCC silver structure. The diffractogram for Pt@RO displayed three peaks at 2θ positions of 39.82°, 46.30°, and 67.55° comparison to the JCPDS reference card for platinum (File No. 04-0802) confirmed that these peaks correspond to the Miller indices (111), (200), and (220) of the FCC platinum structure.

The presence of low-intensity peaks in the diffractograms can be attributed to several factors, including small crystallite size, which causes peak broadening and reduces intensity, as well as poor crystallinity, leading to weaker diffraction signals. Additionally, an insufficient sample quantity may limit the interaction with X-rays, resulting in weak peaks. High background noise from amorphous phases, such as the organic fraction of the RO extract or sample holder scattering, may also overshadow diffraction peaks [37]. An estimation of the crystallite size of the samples was calculated using the Scherrer equation, yielding values of 5.2 nm and 6.5 nm for Au@RO and Ag@RO, respectively. In the case of Pt@RO, the small size of the nanoparticles and the presence of organic matter from the RO extract contribute to broad, low-intensity XRD peaks with high background. Under these conditions, crystallite size calculation using the Scherrer equation would not provide reliable or meaningful values, often yielding widely varying results and potentially under- or overestimating the actual nanoparticle size [38].

The crystallite sizes obtained from XRD are smaller than the particle sizes observed by TEM (7.5 ± 2.3 nm for Au@RO and 12.9 ± 3.5 nm for Ag@RO). This difference is expected, as XRD measures the size of coherent crystalline domains, while TEM provides the overall particle size, including any polycrystalline aggregates.

**Figure 4:**
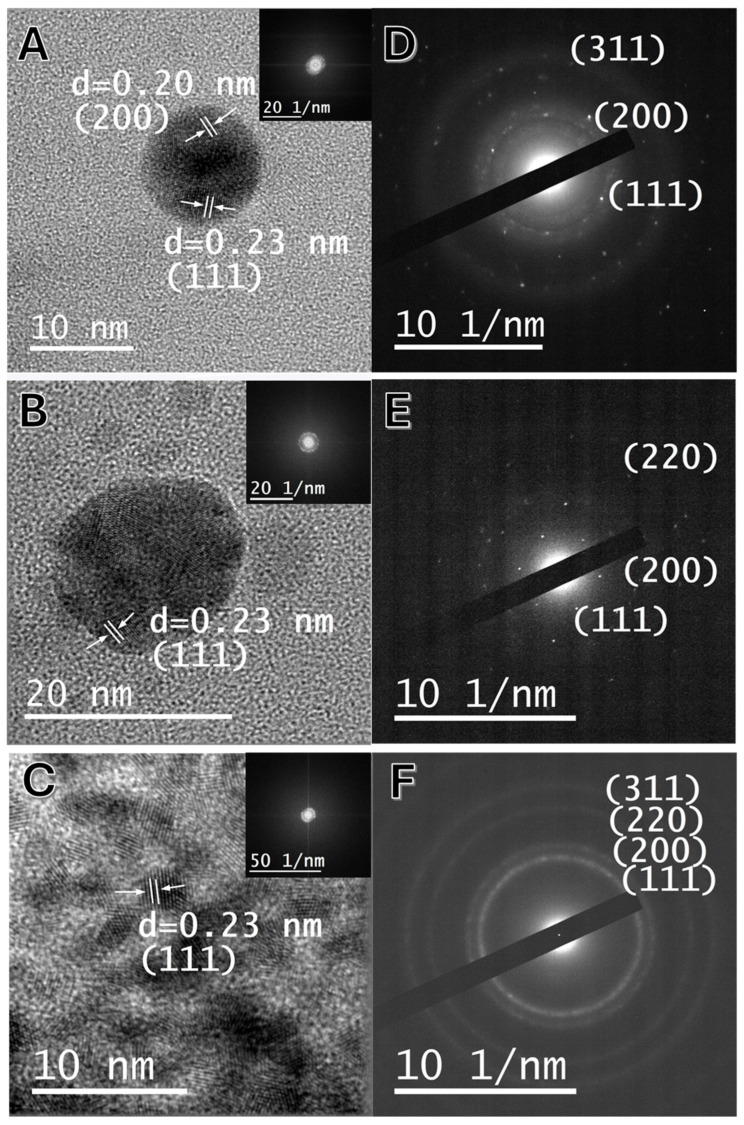
(**A**–**C**) High-resolution images and Fourier transform (inset); (**D**–**F**) selected area diffraction pattern of Au@RO, Ag@RO and Pt@RO, respectively.

Taken together, these observations suggest that the nanoparticles are likely polycrystalline, with each particle composed of several small crystalline domains. Therefore, the slightly larger TEM sizes reflect the full nanoparticle morphology, whereas XRD provides a measure of the internal crystalline domain size. This finding was further corroborated by high-resolution transmission electron microscopy (HRTEM) imaging. Figure 4A,B present the HRTEM images of individual Au@RO and Ag@RO nanoparticles, respectively, while Figure 4C shows a group of small crystalline Pt@RO. In Au@RO and Ag@RO, distinct lattice fringes with different orientations can be observed within a single nanoparticle, indicating multiple crystalline domains. Furthermore, the corresponding fast Fourier transform (FFT) images (insets) appear as a mixture of discrete points rather than a regular pattern, confirming the polycrystalline nature of the samples.

The measured d-spacing values for Au@RO were 0.23 nm and 0.20 nm, corresponding to the (111) and (200) Miller indices, respectively. For Ag@RO, the obtained d-spacing was 0.23 nm, associated with the (111) Miller index and similarly for Pt@RO, where a d-spacing of 0.23 nm associated with the (111) Miller index was also obtained. Figure 4D–F display the selected area electron diffraction (SAED) patterns of a group of Au@RO, Ag@RO and Pt@RO nanoparticles, respectively. These patterns exhibit multiple concentric rings composed of discrete spots, indicating the presence of multiple crystal orientations. In the case of Au@RO, the diffraction spots correspond to the (111), (200), and (311) planes, for Ag@RO, the observed planes include (111), (200), and (220) whereas for Pt@RO the observed planes were (111), (200), (220) and (311).

These complementary analyses, in comparison with tabulated reference data, confirm the polycrystalline nature of Au@RO, Ag@RO and Pt@RO samples, consistent with their FCC crystal structure.

### 2.4. Surface Charge of Au@RO,Ag@RO and Pt@RO

The zeta potential of the synthesized MeNPs was measured to evaluate their surface charge, which is indicative of their stability. The recorded values were −17.6 ± 0.4 mV for Au@RO, −23.9 ± 0.9 mV for Ag@RO and −19.8 ± 0.8 mV for Pt@RO, indicating that the synthesized nanoparticles possess a negative surface charge. According to the literature, zeta potential values between ±20 and ±30 mV indicate moderate stability, while values between ±10 and ±20 mV suggest relative stability, since the presence of these electrostatic forces prevents aggregation and contributes to the stability of the colloidal suspensions [39]. Based on these classifications, Au@RO and Pt@RO exhibit relative stability, whereas Ag@RO demonstrates moderate stability, suggesting their ability to remain dispersed under the tested conditions. Previous studies on the green synthesis of MeNPs from brown macroalgae have reported similar zeta potential values, ranging from approximately −16.9 to −30.6 mV for AuNPs [11,23], −22.6 to −30.0 mV for AgNPs [31,40,41] and −19.9 mV for PtNPs [36]. The lower stability observed for Au@RO and Pt@RO compared to Ag@RO may be attributed to its smaller particle size. The synthesis of MeNPs with diameters below 10 nm while maintaining good stability is particularly challenging due to their high surface energy, which increases their tendency to aggregate. In contrast, larger nanoparticles or those with stronger electrostatic or steric stabilization mechanisms tend to exhibit higher colloidal stability [42]. Despite the inherent challenges, in this study, Au@RO and Pt@RO with diameters below 10 nm and good stability were successfully synthesized.

### 2.5. Fourier Transform Infrared Spectroscopy Characterization

**Figure 5:**
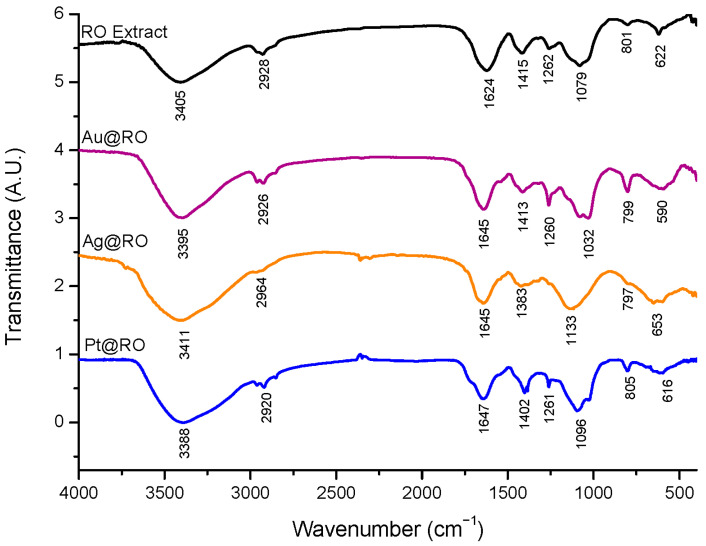
Fourier transform infrared spectra comparing RO extract, Au@RO, Ag@RO and Pt@RO.

Fourier Transform Infrared spectroscopy (FTIR) was performed on RO extract, Au@RO, Ag@RO and Pt@RO to evaluate potential alterations in the functional groups of the extract’s molecules potentially induced by nanoparticle synthesis aiming to explore possible indications of their role in reduction and stabilization processes (Figure 5). The complexity of spectrum interpretation and band assignment increases with the compositional intricacy of the samples. The assignment of bands in the spectra of algae extracts is particularly challenging; therefore, we compared our spectra with previous studies on *R. okamurae* biomass and extracts [43,44,45,46,47,48], as well as our prior research on brown seaweed aqueous extracts [11,22,23].

The RO extract is likely to be rich in water-soluble components of *R. okamurae*. Several studies on *R. okamurae* composition have identified carbohydrates as the major constituents, with lower proportions of proteins and polyphenols [49,50,51,52]. However, discrepancies exist regarding the specific carbohydrate composition. Some studies report that *R. okamurae* carbohydrates primarily consist of cellulose, hemicellulose, and lignin [43,53], whereas others quantify fucoidan, laminarin, and alginates, along with monosaccharides, including glucose, fucose, arabinose, mannitol, galactose, and xylose [51,52]. These variations likely arise from differences in extraction techniques and analytical methodologies.

In general, cellulose could be extracted from residual biomass following the extraction of other components, such as proteins and hydrocolloids, since its insolubility in water enables it to remain intact despite multiple aqueous treatments [54]. Consequently, cellulose is unlikely to be present in the RO extract. Indeed, the FTIR spectrum profile obtained for RO extract (Figure 5) showed similarities with those previously reported for other aqueous algae extracts, where polysaccharides such as fucoidan, carrageenan, or ulvan have been reported [11,55,56]. Typical polysaccharides FTIR spectra are divided into five regions. However, in this case, the position, intensity and broadening of these bands can be altered due to the contribution of other components such as proteins, and polyphenols [57].

The first region of the RO extract spectrum (4000–2500 cm^−1^) exhibited two bands corresponding to functional groups commonly found in macroalgal extracts, which have also been previously observed in *R. okamurae* samples [43,46]. The first prominent and broad absorption band at 3405 cm^−1^ is commonly associated with O-H stretching vibrations, which may indicate the presence of hydroxyl groups typically found in polysaccharides and polyphenols. A second, weaker band at 2928 cm^−1^ corresponds to C-H stretching vibrations, typical of alkane groups. In the second region, between 1800–1500 cm^−1^, an intense band at 1624 cm^−1^ can be observed and has been previously reported as corresponding to carboxylate C-O-C and C=O asymmetric stretching vibration [45,47]. The third region includes the bands between 1500–1200 cm^−1^ that mainly includes deformational vibrations of groups with local symmetry. So, the band at 1415 cm^−1^ can be assigned to the C-O symmetric stretching of carboxylate or to C-OH and -CH_2_ deformation vibrations [43,45]. The region of frequencies between 1200 cm^−1^ and 800 cm^−1^ is called the fingerprint region. In this region, the band at 1262 cm^−1^ has been reported in the literature as potentially related to S=O groups of sulphated polysaccharides such as fucoidan [44,46] although alternative assignments (e.g., glucose ring C=O stretching) have also been suggested [53]. Another intense and wide band is observed at 1079 cm^−1^ which likely corresponds to the C-O and C-C stretching vibrations of the pyranose ring [11,45,58], as well as the C-O-C stretching vibrations characteristic of the glycosidic bonds [43]. Finally, a very weak band is observed at 801 cm^−1^, which is likely associated with a C-O-S vibration [45].

A comparison of the RO extract before and after the synthesis of nanoparticles was performed. In all cases, a notable shift to a higher wavenumber was observed in the band assigned to C=O stretching increasing from 1624 cm^−1^ in the RO extract to 1645 cm^−1^ for both Au@RO and Ag@RO and 1647 cm^−1^ for Pt@RO. Changes in the position were also observed for the band at ~3400 cm^−1^. However, in the case of Ag@RO, notable changes were observed in both the intensity and position of almost all bands. The most prominent difference between Ag@RO and the RO extract is the disappearance of the band at 1262 cm^−1^ while in the case of Au@RO this band appears more intense and sharper. These variations may suggest the possible involvement of sulphated polysaccharides in the reduction and stabilization process, although the participation of other components cannot be excluded [11,22].

### 2.6. Antioxidant Activity

**Figure 6:**
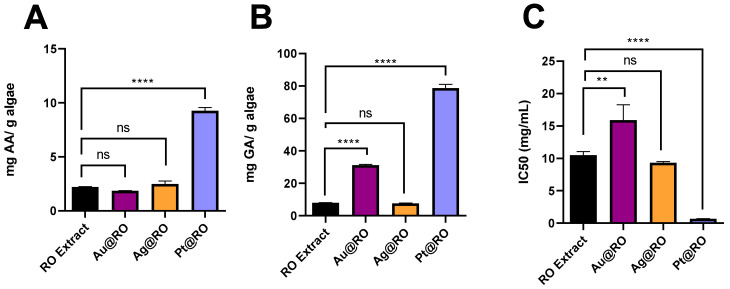
Bar graphs comparing (**A**) reducing activity, (**B**) total phenolic content, and (**C**) DPPH scavenging activity of the RO extract, both before and after the synthesis of Au@RO, Ag@RO and Pt@RO. Statistical significance is indicated as follows: ns, not significant; ** *p* ≤ 0.01; **** *p* ≤ 0.0001.

Figure 6 presents the reducing power, total phenolic content, and 1, 1-diphenyl-2-picryl-hydrazyl (DPPH) radical scavenging activity of the RO extract, which was analyzed both before and after the synthesis of MeNPs. Macroalgae are widely recognized as rich sources of antioxidants [59], and the antioxidant properties of algal extracts play a crucial role in the stabilization and formation of MeNPs [60]. However, studies on the antioxidant properties of different macroalgae species remain particularly scarce, as in the case of *R. okamurae*, making it challenging to draw comprehensive comparisons.

The reducing power value for the RO extract was observed to be 2.2 ± 0.03 mg AA/g dried algae (Figure 6A). Variations in values may arise due to differences in the assay and the aqueous extraction procedures; however, no previous studies were found reporting the reducing capacity of *R. okamurae* using the Oyaizu method. Córdoba-Granados et al. reported the total antioxidant activity of *R. okamurae* using ferric reducing antioxidant power (FRAP) assay obtaining values ranging from 7.9 to 12.5 mg AA/g lyophilized extract [51]. These values are lower than those documented for other brown macroalgae, such as *S. muticum*, *U. pinnatifida and Saccorhiza polyschides*, which showed values of 81.5 ± 2.9 mg AA/g fresh macroalgae, 51.2 ± 1.6 mg AA/g fresh macroalgae and 67.0 ± 1.2 mg AA/g fresh macroalgae, respectively, using the same assay method employed in the present study, although the extraction procedure presented some variances [11,22,23]. Nevertheless, the reducing power present in the RO extract of this study was sufficient to successfully produce MeNPs.

In terms of the total phenolic content of the RO extract, it exhibited a value of 7.9 ± 0.1 mg GA/g algae (Figure 6B), which falls within the range of previously reported values for *R. okamurae* samples obtained using different extraction techniques, varying from 2.7 ± 0.2 mg GA/g dry RO to 12.4 ± 0.7 mg GA/g volatile solids [49,53]. Additionally, previously reported aqueous extracts of *R. okamurae* collected from the same location and using a similar extraction procedure to that in the present study showed total phenolic content values ranging from 3.4 to 6.7 mg GA/g lyophilized extract, which are very similar to those observed in this work. In the above-mentioned study, there were also determined variations in the total phenolic content of *R. okamurae* collected from the same location over different years, which may explain the slight differences compared to the values obtained in the present study [51]. Other changes in physicochemical environmental factors such as salinity, nutrients, light or collection depth can also impact total phenolic content [61]. Previous studies on *R. okamurae* have identified phenolic compounds as the main contributors to the antioxidant activity of RO extracts [49].

The DPPH-free radical scavenging capacity of the RO extract was measured, obtaining an IC50 value of 10.49 ± 0.6 mg/mL (Figure 6C) and 21.1 ± 3.3% in terms of percentage of inhibition (IP) for a concentration of 10 mg/mL. It is important to note that the solvent used for the macroalgae extraction significantly affects the DPPH results [23], making direct comparisons with DPPH values reported for other RO samples in the literature challenging. However, previous studies have indicated that the highest DPPH scavenging values for *R. okamurae* were achieved when water was used as the extraction solvent [52]. In fact, *R. okamurae* samples subjected to an ethanol-water extraction have previously shown IP values ranging from 4.6 ± 0.1 to 6.1 ± 0.1, which are approximately four times lower than the values observed in the present study. However, the concentration of extract used in that assay was not specified. [45]. The DPPH values obtained in the present study are notably lower than those reported for other brown macroalgae using the same DPPH assay, being nearly seven times lower than those of *S. muticum* and eleven times lower than *U. pinnatifida* [11,23]. However, slight differences in the extraction methods may also contribute to the observed variance in the DPPH values among the brown macroalgae.

When comparing the results of the RO extract with those obtained after the synthesis of MeNPs, a significant increase in total phenolic content was observed in Au@RO and Pt@RO, with Pt@RO exhibiting the highest enhancement, reaching values eight times higher than the RO extract. In contrast, Ag@RO did not show significant differences in any of the three assays compared to the extract.

Despite the increased total phenolic content in Au@RO, no significant improvement was observed in its reducing power or DPPH scavenging activity, suggesting that the presence of AuNPs did not enhance the extract’s antioxidant capacity beyond phenolic retention for the specific assay. This aligns with previous reports indicating that while green-synthesized AuNPs can concentrate phenolic compounds, their impact on antioxidant activity varies depending on the specific assay [23]. The lack of improvement in reducing power further supports the idea that this property is primarily determined by the intrinsic characteristics of the RO extract rather than by the AuNPs themselves.

On the other hand, compared to Au@RO and Ag@RO, Pt@RO exhibited significant improvements across all three antioxidant assays, with a fourfold increase in reducing power and a notable enhancement in DPPH scavenging activity. This superior performance is likely due to a combination of factors: the extremely small size of the PtNPs synthesized (2.6 ± 0.8 nm) increases the surface-to-volume ratio, providing more active sites for interactions; the negative zeta potential (−19.8 ± 0.8 mV) may influence the adsorption and orientation of bioactive molecules on the particle surface; and the observed shift in the C=O stretching from 1624 cm^−1^ in the RO extract to 1647 cm^−1^ in Pt@RO (FTIR) suggests interactions between carbonyl-containing bioactive molecules and the PtNPs surface. These interactions could help stabilize the bioactive compounds on the nanoparticle surface, enhancing electron transfer and contributing to the observed increase in antioxidant activity. Together, these factors suggest that both surface chemistry and physical properties of PtNPs enhance the extract’s antioxidant activity. This behavior has been reported in other studies where PtNPs synthesized by green synthesis have been identified as highly effective in increasing the antioxidant activity of the extract [32,62,63].

In contrast, Ag@RO did not exhibit any significant differences in total phenolic content, reducing power, or DPPH scavenging activity compared to the RO extract. This could be attributed to its larger particle size (12.9 ± 3.5 nm) relative to Au@RO (7.5 ± 2.3 nm) and Pt@RO (2.6 ± 0.8 nm), which results in a lower surface-to-volume ratio and fewer active sites available for interactions with bioactive compounds. The lack of enhancement across all assays indicates that Ag@RO, under these synthesis conditions, do not provide additional benefits in terms of antioxidant activity.

Taken together, these results underscore the critical influence of nanoparticle composition and physicochemical properties on antioxidant performance and show that the interaction between the RO extract and the metal nanoparticles differentially affects the antioxidant response, with Pt@RO exhibiting the greatest enhancement across all assays under the experimental conditions used in this work. Such an increase in antioxidant activity of Pt@RO compared with the RO extract alone may be relevant in established applications of Pt nanoparticles where redox or radical-scavenging properties are important, for example, in heterogeneous catalysis and environmental remediation processes operating under oxidative conditions, or in antioxidant coatings and hybrid materials based on Pt nanostructures [19].

## 3. Materials and Methods

### 3.1. Algae Collection and Extract Preparation

*R. okamurae* samples were collected from the lower intertidal zone (swash area) along the coast of Cádiz, Spain (36.185869, −5.946970), in November 2024 by Dr. Ivan Franco Rodil, from the Instituto Universitario de Investigación Marina of Universidad de Cádiz, Spain.

Samples were rinsed on-site with seawater and then transported to the laboratory, where they were washed with distilled water and dried in an oven P Selecta Dry big (JP Selecta, Barcelona, Spain) at 60 °C for four days.

*R. okamurae* samples were transported to the laboratory and stored at −20 °C until processed. When needed, the stored *R. okamurae* was ground into a fine powder. Then, 1 g of powder was added to 100 mL of boiling Milli-Q water and maintained at a boil for 30 min. The mixture was then centrifuged at 4000 rpm for 20 min, the supernatant was filtered and stored at −20 °C for later use.

### 3.2. Synthesis of Gold, Silver, and Platinum Nanoparticles

The synthesis of AuNPs, AgNPs and PtNPs using the previously prepared RO extract was optimized by evaluating a range of extract concentrations and metal salt concentrations, using an aqueous 0.01 M HAuCl_4_ solution for AuNPs (Au@RO), 0.005 M AgNO_3_ solution for AgNPs (Ag@RO), and 0.01 M PtCl_4_ solution for PtNPs (Pt@RO), as precursors. The optimization process also included testing various temperatures and reaction times. UV-Vis spectroscopy was used to evaluate the progress of the reactions, while the formation of nanoparticles was confirmed by TEM.

The procedure can be briefly described as follows: the aqueous extract of the desired concentration was heated under continuous stirring using a round-bottom flask placed on a magnetic stirrer with a built-in heating plate (MR Hei Tec, Heidolph, Schwabach, Germany). Once the extract reached the set temperature, the metal salt solution was slowly added under constant stirring and left to react. Syntheses at high temperatures were performed under open reflux conditions. The optimal reaction conditions obtained are summarized in Table 1.

### 3.3. Characterization Techniques

Before each characterization technique, the RO extract was used directly without additional processing. In contrast, the Au@RO, Ag@RO and Pt@RO nanoparticle suspensions were used as-prepared for UV–Vis, zeta potential, FTIR and antioxidant assays, and were only subjected to a common purification protocol when required for TEM and XRD analyses, as detailed below.

RO extract, Au@RO, Ag@RO and Pt@RO were characterized by UV-Vis spectroscopy using a Jasco V-670 spectrometer (JASCO, Tokyo, Japan) over a wavelength range of 200–800 nm at room temperature. UV-Vis spectra of the RO extract and nanoparticle suspensions were recorded using Milli-Q water as the blank, since it was the solvent in all preparations, ensuring that only sample absorbance was measured.

Au@RO, Ag@RO and Pt@RO samples were purified to reduce the organic fraction for TEM analysis by centrifugation at 10,000 rpm for 30 min at room temperature. The resulting pellets were resuspended in Milli-Q water and sonicated for 15 min to ensure proper dispersion. A volume of 20 µL of the samples was then placed onto 400 mesh copper grids coated with Formvar and carbon. The TEM analysis was carried out with a JEOL JEM 1010 (100 kV) microscope (JEOL, Tokyo, Japan), while HRTEM imaging was conducted with a JEOL JEM2010F field emission gun TEM (200 kV, JEOL, Tokyo, Japan).

Zeta potential was measured using a Zetasizer Nano S (Malvern Instruments, Malvern, UK), with He-Ne laser (wavelength 633 nm) and backscatter detection at 173°. Before the analysis Au@RO, Ag@RO and Pt@RO samples were sonicated for 15 min. The measurement was based on the average of five independent readings.

RO extract and the MeNPs samples were dried in an oven at 80 °C and finely pulverized into a powder to prepare KBr pellets for FTIR analysis, using a Jasco FT/IR-6100 spectrophotometer (JASCO, Tokyo, Japan). The spectra were collected in the 4000–400 cm^−1^ range, with a total of 60 scans conducted at a resolution of 4 cm^−1^ and a scan rate of 2 mm/s.

For the preparation of Au@RO, Ag@RO and Pt@RO samples for XRD, the samples were centrifuged at 10,000 rpm for 30 min at room temperature, the supernatant was removed, and the resulting pellet was resuspended in Milli-Q water. Then, the samples were deposited onto a glass substrate and dried. XRD diffractograms were acquired within the 2θ range of 20–90° with a step size of 0.013° employing a XpertPro diffractometer (Malvern Panalytical, Malvern, UK) with Cu Kα radiation (λ = 0.154 nm). The instrument operated at 40 mV current and 40 kV voltage. The Scherrer equation is used to derive the average crystallite size [64]:

(1) D=(κλ)/(βcosθ),

where D is the crystallite size, κ is a dimensionless shape factor with a value of 0.9 due to the spherical shape of the nanoparticles, λ= 1.54 Å is the wavelength of the X-rays, β is the full width at half maximum (FWHM) of the diffraction peak in the XRD pattern, and θ is the angle at which the maximum peak is observed.

### 3.4. Antioxidant Activity

Antioxidant activity of the RO extract, Au@RO, Ag@RO and Pt@RO, was assessed through three different colorimetric methods: reducing power, total phenolic content, and DPPH radical scavenging activity. These assessments were conducted in accordance with established protocols from previous studies [65,66]. For antioxidant assays, both the RO extract and the nanoparticle suspensions were used directly in their aqueous form, without any additional processing, to preserve their original chemical composition.

#### 3.4.1. Reducing Power

For the assessment of reducing power, 1 mL of the sample (RO extract, Au@RO, Ag@RO, Pt@RO or Milli-Q water in the case of the blank control) was mixed with 2.5 mL of 1% potassium ferricyanide and 2.5 mL of phosphate buffer (0.2 M, pH 6.6). The mixture was incubated for 20 min at 50 °C. The reaction stopped by adding 2.5 mL of 10% trichloroacetic acid. The resulting solution was centrifuged at 3000 rpm for 10 min and 2.5 mL of the supernatant was combined with 2.5 mL of Milli-Q water. Upon adding 0.5 mL of 0.1% ferric chloride, a rapid color change from yellow to blue occurred, and the absorbance was immediately recorded at 700 nm. A calibration curve was prepared using concentrations from 60 to 370 mg/L of ascorbic acid as the standard. Results are presented as the mean of three independent assays and are expressed as ascorbic acid (AA) equivalents per gram of macroalgae.

#### 3.4.2. Total Phenolic Content

A total of 100 µL of the sample (RO extract, Au@RO, Ag@RO, Pt@RO or Milli-Q water for the blank control) was combined with 2 mL of 5% sodium carbonate and incubated for 2 min at room temperature. Then, 100 µL of 50% Folin–Ciocalteu reagent was added and thoroughly mixed. The reaction solution was incubated in the dark at room temperature for 30 min and the absorbance was recorded at 725 nm. A calibration curve was constructed using gallic acid at concentrations between 0.1 and 0.75 mg/mL. All experiments were carried out in triplicate, and the results are reported as gallic acid (GA) equivalents per gram of macroalgae.

#### 3.4.3. DPPH Scavenging Activity

A stock solution of 0.1 mM DPPH was prepared in methanol. Then, 1 mL of this solution was combined with 3 mL of sample (RO extract, Au@RO, Ag@RO or Pt@RO). A blank was prepared by replacing the sample with 3 mL of Milli-Q water, while a sample control was prepared by mixing 3 mL of sample with 1 mL of methanol as a substitute for the DPPH solution. All mixtures were allowed to react at room temperature for 30 min. After this incubation period, absorbance was read at 517 nm. The DPPH radical scavenging activity was determined using the formula:

$$DPPH\;scavenging\;effect\left(\%\;inhibition\right)=\left(1-\frac{A_{s}-A_{s0}}{A_{b}}\right)\times 100$$

where A_s_ is the absorbance of the sample, A_s0_ is the absorbance of the sample control, and A_b_ is the absorbance of the blank.

Each sample was tested three times, and the results are presented as the concentration needed to reduce the DPPH radical concentration by 50% (IC50).

#### 3.4.4. Statistical Analysis

The experimental data were processed and graphed using SPSS software version 23.0 and GraphPad Prism 9, with results expressed as mean values ± standard deviation (SD). Data were tested for normality using the Shapiro–Wilk test and for variance homogeneity with Levene’s test. After the confirmation of normality assumptions were met, one-way analysis of variance (ANOVA) was then performed followed by Dunnett’s post hoc test, used to identify significant differences between RO extract and Au@RO/Ag@RO/Pt@RO. A significance threshold of 0.05 was adopted for all analyses: * *p* ≤ 0.05, ** *p* ≤ 0.01, *** *p* ≤ 0.001, and **** *p* ≤ 0.0001.

## 4. Conclusions

This study successfully demonstrates the potential of *R. okamurae* to act as a sustainable reducing and stabilizing agent for the synthesis of gold, silver, and platinum nanoparticles. The results highlight the efficiency of *R. okamurae* extract in providing an eco-friendly alternative to conventional synthesis methods that often rely on toxic chemicals and energy-intensive processes. The optimal conditions for nanoparticle synthesis led to the formation of stable, spherical and polycrystalline particles of 7.5 ± 2.3, 12.9 ± 3.5 and 2.6 ± 0.8 nm for Au@RO, Ag@RO and Pt@RO, respectively. Among the synthesized nanoparticles, Pt@RO exhibited the highest antioxidant activity, significantly increasing the total phenolic content (31.18 ± 0.45 mg GA/g) and reducing power (8.8 ± 0.6 mg AA/g) compared to the RO extract. Additionally, Pt@RO nanoparticles showed the greatest improvement in DPPH radical scavenging activity, with an IC50 value of 5.2 ± 0.4 mg/mL, highlighting their potential biomedical applications. Furthermore, this study offers a novel approach to mitigating the environmental impact of *R. okamurae* by transforming an ecological threat into a valuable resource.

## Figures and Tables

**Table 1:** Optimal reaction conditions for Au@RO, Ag@RO and Pt@RO synthesis.

Code	[Extract] (mg/mL)	[Au] (mM)	[Ag] (mM)	[Pt] (mM)	T (°C)	t (h)
Au@RO	6.67	0.33	-	-	30	24
Ag@RO	10	-	0.17	-	100	2
Pt@RO	1	-	-	0.4	100	3

## Data Availability

The data supporting the findings of this study are available within the article and its ESI.

## Supplementary Materials

The following supporting information can be downloaded at https://www.mdpi.com/article/10.3390/md23120479/s1, Figure S1: UV Spectra of gold NPs synthesized with varying gold concentrations (0.17–0.5 mM) and different concentrations of RO extract: (A) 10 mg/mL, (B) 6.67 mg/mL, and (C) 5 mg/mL; Figure S2: TEM images of gold NPs synthesized using 3.3 mg/mL of RO extract, 0.42 mM Au, at 30 °C for 24 h.
