# Supplementary material for: Exploiting the Invasive Alga Rugulopteryx okamurae for the Synthesis of Metal Nanoparticles and an Investigation of Their Antioxidant Properties
Source: Mar Drugs. 2025 Dec 15;23(12):479. doi: 10.3390/md23120479 (PMC12734819; doi:10.3390/md23120479)
Supplement: Supplementary file 1 [file marinedrugs-23-00479-s001.zip › marinedrugs-3899479-supplementary.pdf]

Supplementary Material

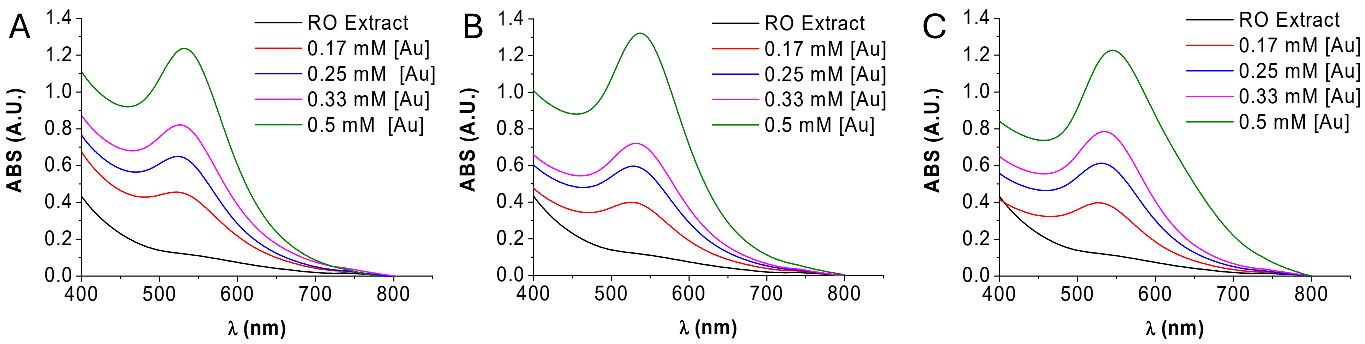

Figure S1. UV Spectra of gold NPs synthesized with varying gold concentrations (0.17–0.5 mM) and different concentrations of RO extract: (A) 10 mg/mL, (B) 6.67 mg/mL, and (C) 5 mg/mL.

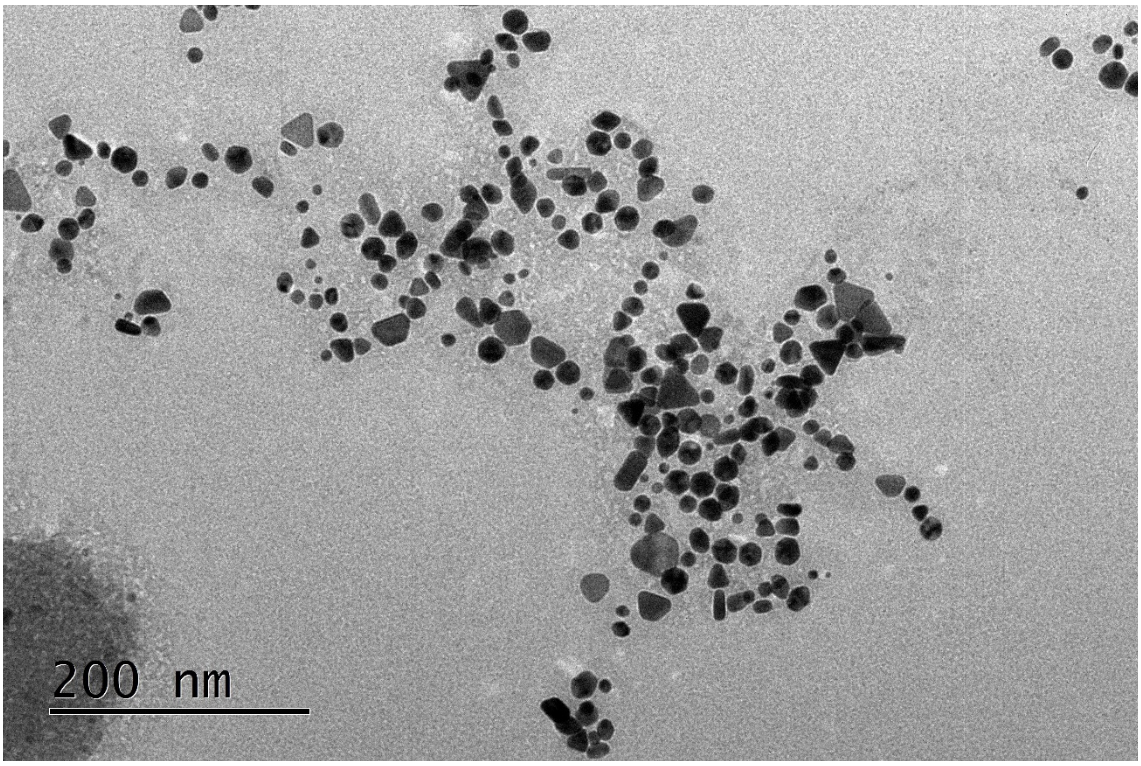

Figure S2. TEM images of gold NPs synthesized using 3.3 mg/mL of RO extract, 0.42 mM Au, at 30 ºC for 24 hours.
